# The Efficacy of *Passiflora Incarnata Linnaeus* in Reducing Dental Anxiety in Patients Undergoing Periodontal Treatment

**Published:** 2013-06

**Authors:** N Kaviani, M Tavakoli, MR Tabanmehr, RA Havaei

**Affiliations:** a Dept. of Oral & Maxillofacial Surgery and Torabinejad Dental Research Center, School of Dentistry, Isfahan Medical University of Medical Sciecnes, Isfahan, Iran.; b Dept. of Periodontology and Torabninejad Dental Research Center, School of Dentistry, Isfahan Medical University of Medical Sciecnes, Isfahan, Iran.; c Dentist; d Dental Student

**Keywords:** Dental Anxiety, Passion flower (Passiflora Incarnata L.), Premedication

## Abstract

**Statement of Problem: **Oral premedication used to reduce the anxiety in patients undergoing dental treatment. Passion flower has been used as a sedative that can control the dental anxiety.

**Purpose:** This study determines the efficacy of Passion flower, in reducing anxiety during the dental procedures.

**Material and Methods:** In this randomized- one sided blind clinical trial, 63 patients, with moderate, high and severe anxiety(according to VAS score) in need of periodontal treatment were randomly divided into 3 groups of 21.The first group was given the drop Passion flower drop and the second group were given the drop of placebo and the third group; neither drug nor placebo were given (negative control group). Results were analyzed by Chi Square, Variance Analysis, Tucky and Paired-T using SPSS software.

**Results:** Mean anxiety level prior to the drug administration was 12.09±2.42 for the Passion flower group, 12.00±2.66 for the placebo group and 11.66±2.39 for the negative control group. After premedication, these values were: 8.47±2.58 for the Passion flower group, 10.52±2.11 for the placebo group and 11.23±2.34 for the negative control group. These results demonstrated a significant difference (*p*< 0.0001) in the anxiety levels before and after the Passion flower administration in the Passion flower group and also between the Passion flower group and the other two groups.

**Conclusion:** Results indicated that administration of Passion flower, as a premedication, is significantly effective in reducing the anxiety. Since this study is a pioneer on the subject, further trials with greater number of subjects are required to confirm our results.

## Introduction

Dentistry- related anxiety is a problem that many patients all around the word may face and is considered as an important issue in dental and oral health care services. As a main discourager in seeking dental treatments; anxiety may lead to a poor oral health and other psycho-social problems in addition to the problematic dental treatments for the dentist. Controling the anxiety during the treatment is therefore a crucial element for both dentist and patient [1-5]. 

Oral premedication is a simple effective method for anxiety control. Various drugs are used including benzodiazepines, antihistamines for this purpose. Herbal drugs with sedative effects, such as Passion flower, have been used throughout the history as the anti-anxiety and sedative drugs [6-8].

Passion flower, *Passiflora Incarnata Linneaus*, is a dynamic climbing plant which utilizes tendrils in its upward growth. It has woody stems reaching up to 8-9 meters. All parts (flowers, leaves, and stems) of the Passion flower are  used for medicinal purposes. Its usage suppresses the nervous system; leading to a state of peace [9-13]. This plant is listed in the Martindale (The Extra pharmacopoeia) as a sedative, anti-spastic drug used in various compound drugs in 24 different countries[10]. Its application in controlling the anxiety has been confirmed in various authentic institutions including Scop, British herbal compendium (Passionflower Herb, England), German commission, standard licences (*Passiflora incarnate,* Germany) [9-13].

In Iran; its extract has been introduced to the market by the pharmaceutical company ‘’Darook’’ in the forms of drops and tablets with the trade name ‘’Pasipi’’, which has been examined in various researches in different parts of Iran. The pharmaceutical company ‘’Niak’’ also uses this plant in a compound drug called Vliflur which has sedative applications [11-15].

The sedative effects of Passion flower was assessed in a cohort randomized controlled study enrolled by Vazirani et al. in 2001 [14]. In this study a total of 36 patients with general anxiety disorder were categorized into two groups. One group recieved 45 drops of Passion flower extract and the other group received Oxazepam tablet 30 mg on a daily basis. Results showed Passion flower to have similar sedative effects as Oxazepam butwith less occupational difficulties which indicates a higher priority of Passion flower to Oxazepam [14].

In another cohort study by Movafagh et al. in 2008; Passion flower extract was used as a premedication for patients who were scheduled for hernia surgery. A total of 60 patients were randomized into two groups with one receiving Passion flower tablets (500 mg) and the other administered with placebo tablets.

Anxiety levels were measured using Numeric Rating Scale (NRS) prior to the administration of medication and 10, 30, 60 and 90 minutes following drug administration. Results indicated that anxiety levels were significantly lower in the group receiving Passion flower compared to the placebo group (*p*< 0.001) [11].

Using this plant, as sedative in dentistry, is tested and encouraged for the first time. Results of this study may help develop various herbal drugs for anxiety control in dentistry. Since most people prefer herbal medications over chemical drugs [16]; hoping to have higher psychological acceptance.

In addition, this research may provide an insight into the medical properties of various herbs and since this drug (Passion flower) is available in the market; these studies may pave the way for developing similar medications with lower costs, reduced side effects and higher availability [17].

## Material and Methods

A total of 63 adult patients compatible with ASA class I & II classification, were selected from the patients admitted to the periodontal department of Isfahan dental school for periodontal surgery. After thorough explanation on the type and method of the study; a written consent was obtained and the patients were randomized into 3 groups as follows:

1. Group administered with Passion flower: drops of Passion flower extract (Pasipi drop, a product of Iran Darook Pharmaceutical Company) were used as premedication and administered to the patients: 20 drops on the night prior to treatment and 20 drops on the morning following treatment (at least 90 min before treatment).
2. Placebo group: placebo was administered as the premedication before treatment in the same way as the first group. Placebo was made by pharmaceutical faculty with no ingredient of any drug or sugar.
3. Groupwith no administered of drugs or placebo (negative control)

All patients completed the Corah’s DAS-R questionnaire once at the time of interview and once following the drug intake and before periodontal treatment. The data were analyzed using SPSS software.

## Results

Subjects included 24 male (38.1%) and 39 female (61.9%) individuals (Table 1). Chi square test showed no significant difference in gender predilection between different groups (*p*= 0.817). With a mean age of 34.07±11.66; the minimum and maximum age of subjects were 18 and 59 years old respectively (*p*= 0.739).

Mean anxiety scores (according to Corah’s DAS-R scale) were found to be 13.09±2.42 in the group receiving Passion flower, 12.00±2.66 in the group receiving placebo, 11.66±2.39 in the negative control group and finally 11.92±2.39 for the whole sample.

**Table 1:** Frequency distribution of samples according to sex

**Group**	**Male**		**Female**		**Total**	
	**Number**	**%**	**Number**	**%**	**Number**	**%**
Passion	8	38.1	13	61.9	21	100
Placebo	9	42.9	12	57.1	21	100
No placebo	7	33.3	14	66.7	21	100
Total	24	38.1	39	61.9	63	100

**Figure 1:**
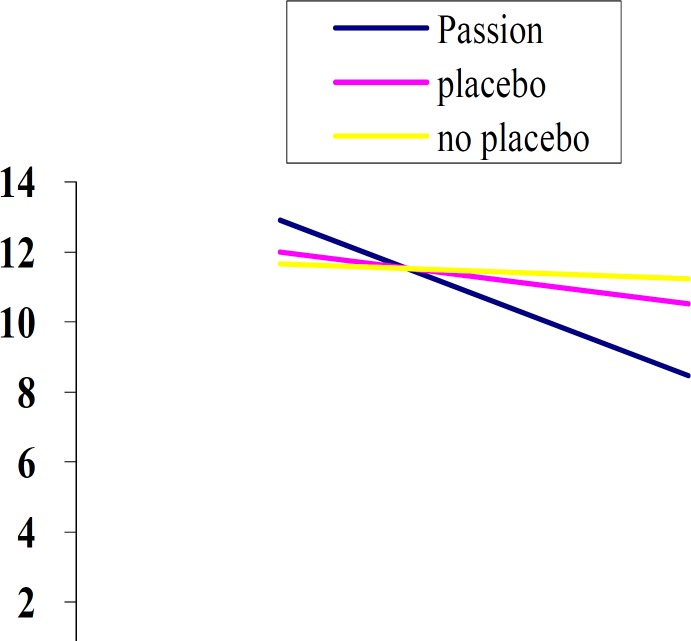
The mean scores of anxiety before and after drug

In the total sample; the minimum and maximum anxiety scores were reported to be 9 and 17 respectively (Table 2 & Figure 1).Variance analysis revealed no significant difference between the 3 groups (*p*= 0.844) in the mean anxiety score before the medication intake.

**Table 2:** Mean anxiety scores for each group before drug

**Group**	**Number**	**Max**	**Min**	**Mean**	**SD**
Passion	21	16	9	12.09	2.42
Placebo	21	17	9	12	2.66
No placebo	21	16	9	11.66	2.39

Mean anxiety scores following the medication intake were shown to be 8.47±2.08 in the group receiving Passion flower, 10.52±2.11 in the group receiving placebo, 11.23±2.34 in the negative control group and finally 10.07±2.45 for the total sample (Table 3). 

**Table 3:** Mean anxiety scores for each group after drug use

**Group**	**Number**	**Max**	**Min**	**Mean**	**SD**
Passion	21	12	5	8.47	2.08
Placebo	21	14	7	10.52	2.11
No placebo	21	17	7	11.23	2.34

Variance analysis indicated significant difference in the mean anxiety score between the groups (*p*= 0.001) which accounts for the difference in at least two groups.

Tukey's Post Hoc test was adopted which found a significant difference between all groups except the placebo group and no- drug group. Moreoever, a significant difference was observed between the Passion flower group and no-drug group (0.010) whereas no difference was found between the placebo group and the negative control group (*p*= 0.543).

Paired t-Test results indicated that the mean anxiety scores before and after the medication intake was significantly different in the group taking Passion flower (*p*< 0.001). This finding was also true in the placebo group (*p*< 0.001) whereas the negative control group did not display any significant difference (*p*= 0.341) (Figure 1).

Variance analysis revealed a significant difference between the mean anxiety scores of before and after the medication intake and between the group taking Passion flower and the placebo group (*p*< 0.001) and also between the group taking Passion flower and the negative control group (*p*< 0.001).

Although no significant difference was found between the placebo group and the negative control group.

## Discussion

The aim of the present study was to determine the effects of Passion flower extract in reducing anxiety in patients undergoing dental treatment.

Dentistry- related anxiety is associated with poor oral and social hygiene for the patient [3] and numerous problems for the clinician during treatment [5]. Premedication is a method that have been used for reducing this anxiety. Passion flower has been widely used in herbal medicine to relieve anxiety in various parts of the world for centuries. Since most people prefer herbal medications over chemical drugs, the use of this medication is believed to be more efficient in reducing patient’s anxiety [16].

As far as the authors are concerned, this is the first study on Passion flower with this purpose of usage a with having both placebo control and negative controlgroup.

Patients in the different groups did not exhibit any significant difference in terms of age and gender (Tables 1). Mean anxiety scores prior to medication intake was also similar in different groups , having no significant differences observed.

Therefore it can be concluded that this therapeutic product accounts for the differences in the mean anxiety scores prior and following drug intake within each group and also between the groups.

A major finding of this research was that Passion flower extract is extremely effective in relieving dental anxiety in patients. The latter is confirmed by statistical analysis which had the following results: The mean anxiety score prior and following drug intake was significantly different in the group receiving Passion flower extract.

Variance analysis indicated that the differences in mean anxiety scores prior and following drug intake between the group taking Passion flower and the placebo group and also between the group taking Passion flower and the negative control group was significantly different. As mentioned previously, this is the first research on the application of Passion flower in reducing dental anxiety. The closest research on the subject was conducted by Movafagh et al. in 2008 [11]. In this study Passion flower was used as a sedative for patients undergoing hernia surgery. Results showed significant reduction in anxiety in the group taking Passion flower compared to that of the placebo group [11].

Our study confirms the latter results as the mean anxiety scores prior and following drug intake were found to be 3.611±.65 in the Passion flower group, 1.47±1.36 in the placebo group and finally 0.42±2.01 in the negative control group. Stated otherwise, anxiety levels in the Passion flower group were significantly lower than the placebo (*p*< 0.001) and the negative control groups (*p*< 0.001).

Consistent with the results of Movafagh et al. [11], our study also showed significant differences in mean anxiety scores prior and following medication intake in both the Passion flower group and the placebo group, which indicates the psycho-somatic effects of the placebo. Moreover, comparison of the differences in the mean anxiety scores prior and following medication intake in the group taking Passion flower and the group taking placebo indicates a significant difference in these values between the two groups. The latter indicates a higher reduced anxiety level in the group taking Passion flower compared to that of the placebo group.

 Mean anxiety score was measured using the Corah’s DAS-R questionnaire and was found to be 10.33 in men and 12.89 in women which was significantly different (*p*< 0.001). Study was performed only on periodontal patients and evaluated the preoperative anxiety. 

 Our results suggest that Passion flower extract is highly effective in reducing dental anxiety. However, being the first study on the subject, more research with bigger samples are required to confirm the results of our study.

Moreover, it is advisable to apply various doses of the medication and study the potential effects.

 Considering the importance of anxiety control in dental patients and abscence of side effects and relatively easy use, other herbal medications are also suggested to be tested for this purpose.

Taking in mind the ever growing use of herbal medications among patients, comprehensive programs for increasing the dentist’s knowledge and awareness of the various properties of herbal drugs is warranted.

## Conclusion

 Passion flower extract, as a sedative agent, is significantly effective in reducing dental anxiety. Since most people have a better opinion towards herbal medications; our goal of reducing anxiety was more readily and accurately achievablewhich makes it more acceptable for both patient and dentist.
